# Reactive infectious mucocutaneous eruption: an increasingly encountered complication during periods of increased *Mycoplasma pneumoniae* transmission—a case report

**DOI:** 10.1128/asmcr.00107-25

**Published:** 2025-09-12

**Authors:** Andrew E. Clark, Lakshmi Marimuthu, Anna Sick-Samuels, Heba H. Mostafa

**Affiliations:** 1 Department of Pathology, Division of Medical Microbiology, Johns Hopkins School of Medicine 1500 , Baltimore, Maryland, USA; 2 Department of Pediatrics, Division of Pediatric Infectious Disease, Johns Hopkins School of Medicine 1500 , Baltimore, Maryland, USA; 3 Department of Hospital Epidemiology and Infection Control, Johns Hopkins Hospital 588543, Baltimore, Maryland, USA; Pattern Bioscience, Austin, Texas, USA

**Keywords:** *Mycoplasma pneumoniae*, RIME

## Abstract

**Background:**

*Mycoplasma pneumoniae* is a common cause of community-acquired pneumonia among children and young adults. Surveillance within the United States has identified increased levels of *M. pneumoniae*-mediated respiratory disease following a multi-year period of minimal detection during the COVID-19 pandemic. A wide spectrum of extrapulmonary complications can be associated with *M. pneumoniae* infections, including dermatological disease. Reactive infectious mucocutaneous eruption (RIME) is a constellation of immune-mediated extrapulmonary manifestations causing ulceration of multiple mucous membranes that occurs in up to 25% *M*. *pneumoniae* cases.

**Case Summary:**

An adolescent patient presenting with acute hypoxic respiratory failure and an eight-day history of conjunctivitis and oral ulcers tested positive for *M. pneumoniae* from a nasopharyngeal specimen and was diagnosed with RIME. A retrospective review of increased *M. pneumoniae* test positivity and hospitalizations at our institution was undertaken, which mirrored national trends.

**Conclusion:**

As dedicated *M. pneumoniae* testing may not be available in all settings, clinicians should consider a diagnosis of RIME for patients presenting with similar para- or post-infectious mucositis, even in the absence of traditional respiratory symptoms. RIME incidence is expected to increase in the setting of a higher prevalence of *M. pneumoniae* disease. As RIME can be associated with significant morbidity including dehydration and weight loss as well as disease recurrence, proper management of patient symptomatology is essential.

## INTRODUCTION

Community-acquired pneumonia (CAP) is a common cause of hospitalization among children and young adults. Prior to the COVID-19 pandemic, respiratory viruses and *Mycoplasma pneumoniae* were among the most common etiologies of CAP detected among hospitalized children (1). Due to the implementation of non-pharmaceutical interventions to reduce SARS-CoV-2 transmission, *M. pneumoniae* almost completely disappeared and did not reemerge to pre-pandemic levels until autumn 2023, one to two years later than other CAP-associated pathogens including respiratory syncytial virus, influenza A, and *Streptococcus pneumoniae* (2). In 2024, the Centers for Disease Control and Prevention (CDC) identified increasing rates of *M. pneumoniae* disease and diagnostic assay positivity among US children (3), a trend that has continued to be observed across the country and in multiple health systems since that time (4).

CAP caused by *M. pneumoniae* infection is often associated with a milder respiratory symptomatology than other etiologies. Sometimes referred to as “walking pneumonia”, these indolent cases still exhibit radiographic findings consistent with pneumonia and can have protracted duration of symptoms (5). A one-to-four-week incubation period is observed prior to symptom onset, which likely contributes to cyclic outbreaks every 3–5 years, thought to be related to population immune status (6, 7). The disease primarily affects children and adolescents, with peak incidence between 5 and 15 years of age, and most commonly presents as a combination of upper respiratory tract infection and tracheobronchitis (8).

*M. pneumoniae* can also cause extrapulmonary disease including immune-mediated mucocutaneous manifestations in about 25% of cases (9). Previously named *Mycoplasma*-induced rash and mucositis (MIRM), this syndrome was recently renamed reactive infectious mucocutaneous eruption (RIME) to account for additional infectious etiologies causing similar presentations (10), as well as to distinguish it from non-infectious dermatologic manifestations with similar pathological appearance including Stevens-Johnson syndrome (SJS) and erythema multiforme (11). RIME primarily affects children and adolescents (5–12 years) with male predominance and is characterized by mucositis of ≥2 surfaces (including conjunctivitis and urethral involvement), infectious prodrome, limited skin involvement, and rule out of an adverse drug reaction (12, 13). Importantly, recurrent episodes of RIME have been estimated to occur in approximately 8–38% of *M. pneumoniae* infections (13, 14).

Despite a low mortality rate, RIME can cause significant morbidity due to interference with oral intake leading to weight loss and dehydration, as well as psychological impacts due to changes in physical appearance secondary to inflammatory symptomatology (10, 13). Here, we describe a case of RIME in an adolescent patient in the setting of increased national rates of *M. pneumoniae* infections compared with previous years. We also examine local rates of *M. pneumoniae* detection and hospitalization at our institution and discuss diagnostic considerations for the detection of *M. pneumoniae*.

## CASE PRESENTATION

**Fig 1:**
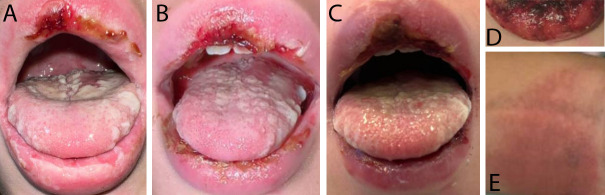
Longitudinal presentation of oral mucosal lesions and associated rash during the patient’s hospital course. Hospital Day 0 (**A**), day 1 (**B**), day 2 (**C**), and Day 6 (**D**). Rash on patient’s right wrist on Hospital Day 2 (**E**).

An otherwise healthy, fully vaccinated adolescent patient presented with respiratory distress in the setting of acute respiratory failure, severe conjunctivitis, oropharyngeal edema, and ulceration (Fig. 1). During the eight days prior to presentation, the patient experienced recurrent fever, sore throat, oral ulcers and sores, cough, wheezes, and tachypnea. Given concern for streptococcal pharyngitis and HSV stomatitis, the patient was treated with amoxicillin and acyclovir at an outside urgent care. The patient continued to experience worsening symptoms and was referred to the emergency department at our institution by the pediatrician due to notable hypoxia and respiratory distress. The patient was assessed in the emergency department where they presented with a swollen mouth with purulent lesions on the lips, tongue, and buccal area, with scabbed lesions on the upper lip, cracked mucosa, and hemorrhagic crusting (Fig. 1A through D). Differential diagnosis included sepsis, myocarditis due to tachycardia and respiratory symptomatology, and SJS, although this was eventually excluded as the mucosal symptoms started prior to beginning any medications other than fever reducers

Given the acuity of the presentation, the patient was transferred to the pediatric ICU where they required critical care due to acute hypoxic respiratory failure. A CT scan revealed narrowing of the nasopharyngeal airway, thickening of the aryepiglottic folds, and patchy opacification of the left lung. In addition to severe oral mucositis, the patient also presented with genital mucositis and minimal skin involvement in the form of mild macular rash (Fig. 1E). The patient also had significant bilateral conjunctivitis and exudate. Upon exam, the conjunctivae were mildly injected, and periorbital swelling and conjunctival erosions were observed. The patient was treated with an amniotic membrane graft placed on both eyes. Based on these findings, the differential diagnosis was narrowed to RIME versus other upper airway infections including retropharyngeal abscess, epiglottitis, tracheitis, or Ludwig’s angina.

A nasopharyngeal swab collected at admission for respiratory molecular testing returned positive for *M. pneumoniae*; no other bacterial or viral respiratory pathogens were detected. Given the identified bacterial etiology and the mucosal surfaces impacted, the patient was diagnosed with RIME secondary to *M. pneumoniae* infection. The patient was managed on a 10 day course of doxycycline (2.2 mg/Kg BID) and systemic steroids (1 mg/Kg/dose, four times per day). Their hospital stay also required significant respiratory support that included high-flow nasal cannula 20L 100%, bilevel positive airway pressure 20/10, and humidified mask, as the patient was not relatively stable on room air until two days prior to discharge.

## DISCUSSION

*M. pneumoniae* is a well-recognized cause of CAP, responsible for up to an estimated 40% of pediatric and 20% of adult cases (15). Transmission is mediated through airborne droplets. While both upper and lower respiratory tract infections are common, definitive diagnosis can be challenging due to nonspecific symptomatology. It is estimated that among children, 10–40% of *M. pneumoniae* respiratory infections will develop into pneumonia (16). While infection is generally self-limiting and may not require antibiotic therapy, azithromycin or a tetracycline remain the drugs of choice for severe infections. Recently, extensive macrolide use has revealed steadily increasing levels of resistance to this drug class. Interestingly, infections with resistant strains do not necessarily correlate with worse clinical outcomes but have been associated with more severe symptomatology and possibly with extrapulmonary complications (17). *M. pneumoniae* infection is also associated with several sequelae, including neurological, hepatic, cardiac, and renal complications that may present following resolution of active infection (18). Cutaneous manifestations, including nonspecific exanthema, urticaria, and RIME, can occur in up to 25% of patients (9).

Accurate estimation of circulating *M. pneumoniae* disease levels has been challenging due to a lack of widely available and timely diagnostic testing in the routine setting (Table 1). Culture is not currently recommended for diagnostic purposes. Serological diagnosis is of retrospective value, with a four-fold increase in *M. pneumoniae* IgG titers between acute and convalescent specimens confirming infection. IgG is usually undetectable in the first week of illness and reaches peak titers 5 weeks after the onset of clinical symptoms. By contrast, IgM antibodies appear within a week, are inconsistently produced among adults but have higher diagnostic value in pediatric populations. Importantly, the historical use of cold agglutinin detection is now considered unreliable due to issues with sensitivity and specificity, as not all patients with *M. pneumoniae* infections elaborate these autoantibodies, and other viral and bacterial pathogens in addition to some malignancies and autoimmune disorders can elicit positive responses (19).

**TABLE 1:** Laboratory diagnostic approaches for *M. pneumoniae* in clinical material

Assay type	Specimen	Timing	Comments
Culture	Respiratory: oropharyngeal/nasopharyngeal swabs, lower respiratory samples (sputum, bronchoalveolar lavage); CSF; sterile body fluids; tissue	First 2 weeks after the onset of illness	Historical gold standard; insensitive; growth takes 21 days or longer; the organism is labile and requires proper transport; technically complex; performed by specialized reference laboratories
Serology	Serum	Antibodies detected after 1 week from the onset of illness, peaking at 3–6 weeks, then gradually declining; paired acute and convalescent sera are preferred (2–3 weeks apart)	Cold agglutinin testing is not recommended; IgM can be detectable for several weeks or months
Molecular detection	Respiratory: oropharyngeal/nasopharyngeal swabs, lower respiratory samples (sputum, bronchoalveolar lavage); CSF: questionable clinical utility; other specimen types: specialized reference laboratories	First 2 weeks after the onset of illness	Multiple FDA-cleared tests are available, including multiplex respiratory panels; molecular prediction of antimicrobial resistance is possible through sequencing

Molecular approaches offer increased sensitivity for detection, and the COVID-19 pandemic facilitated broader access to various molecular modalities allowing for routine *M. pneumoniae* detection in diverse clinical settings. Importantly, while disease is not reportable to health authorities, the expanded use of syndromic panel testing and associated aggregated data have allowed for efficient tracking of *M. pneumoniae* detection levels. Using such aggregate data from the National Syndromic Surveillance Program, the US CDC identified a significant increase in *M. pneumoniae* infections among children, beginning with an increase in late spring and peaking in August 2024. Cases during this time were associated with increases among all ages, with the most pronounced increase observed among young children (3). It is important to note that as *M. pneumoniae* can be detected in the airways of asymptomatic individuals (19), testing in these circumstances may be enriched for patients with severe disease as opposed to outpatients with mild symptomatology, and as *M. pneumoniae* co-circulates with other important CAP-associated pathogens, patient-level interrogation of such cumulative testing data (i.e. in the setting of hospitalizations, etc.) is important (4).

**Fig 2:**
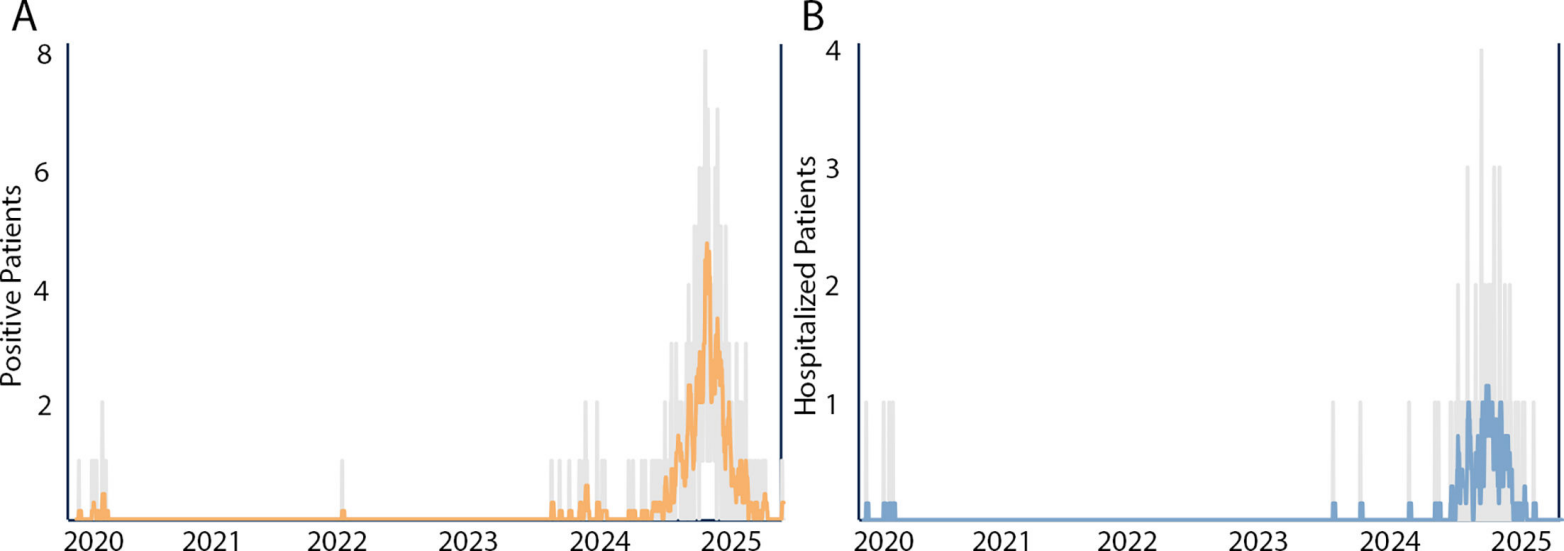
*M. pneumoniae* positive patients (**A**) and associated hospital admissions (**B**) within the Johns Hopkins Health System between 1 January 2020 and 21 May 2025. Colored lines indicate the running 7-day average, and the background gray bars indicate daily cases. Dashboard: Johns Hopkins University.

National *M. pneumoniae* case levels continued to be elevated through spring 2025. To determine if our local case levels mirrored this increase, a retrospective analysis of positive *M. pneumoniae* testing and associated hospitalizations between the years 2020 and 2025 was undertaken. Similar to reported national trends, *M. pneumoniae* detection (Fig. 2A) and associated hospitalizations (Fig. 2B) were almost uniformly absent during the COVID-19 pandemic, with reemergence coinciding with the timeline described in the CDC report (3, 7). *M. pneumoniae*-associated RIME cases were also unreported between 2020 and mid-2024, with additional cases identified during the timeframe which correlated with *M. pneumoniae* re-emergence and the observed increase in hospitalizations. This illustrates that with increased levels of *M. pneumoniae* clinical disease, outbreaks of associated RIME can be expected (20).

Here we present a case of RIME in the setting of a positive respiratory molecular test for *M. pneumoniae*. While in this instance, the identification of the cause of the patient’s symptoms was relatively straightforward, recognition of RIME can be challenging due to its similarity to other skin manifestations including Stevens-Johnson Syndrome and erythema multiforme, which are distinct from RIME as non-infectious sequelae caused by adverse drug reactions. This distinction can be further hampered due to a lack of testing for *M. pneumoniae* or other infectious etiologies (i.e. *Chlamydophila pneumoniae*, viral etiologies) antecedent to presentation, or when respiratory symptoms are lacking.

Despite the well-established association with prior infection, the immunological mechanisms underpinning RIME remain poorly characterized. It is currently unclear if the development of mucocutaneous disease is caused by host or bacteriological factors, or a combination of both (21). Proposed pathophysiologic mechanisms include the involvement of immune complex deposition and complement activation in mucosal tissues due to B-cell clonal expansion and antibody production during *M. pneumoniae* infection. This may be perpetuated by molecular mimicry between *M. pneumoniae* adhesin molecules with structural similarity to keratinocyte antigens leading to immunological cross-reactivity with either antibodies or associated T-cell responses (22). RIME lesions share histopathological characteristics with lesions caused by erythema multiforme and SJS, and detection of *M. pneumoniae* in these lesions is inconsistently reported (23–25).

While prophylactic antibiotic management is of limited use in managing RIME symptomatology unless an active infection is established as in this case, clinical response is largely achieved through the administration of systemic steroids or other immunomodulatory therapy and supportive care (12). Given the possibility of recurrent disease, morbidity associated with the eruptive lesions, and psychological stress associated with the physical manifestations of RIME, it is important to consider this entity, particularly in the setting of increased levels of *M. pneumoniae* disease transmission when managing patients with compatible symptoms, even in the absence of active respiratory symptoms.
